# Case report: preoperative radiological suspicion of atypical meningioma: A diagnostic challenge

**DOI:** 10.1016/j.radcr.2025.12.001

**Published:** 2026-01-06

**Authors:** Raisa Mahmudah, Namira Ammarin, Tasiya Ocvianty

**Affiliations:** Department of Radiology, Faculty of Medicine, Universitas Padjadjaran, West Java, Indonesia

**Keywords:** Atypical meningioma, Neuroimaging, MRI, CT, Diffusion-weighted imaging, Perfusion-weighted imaging, Heterogeneous enhancement

## Abstract

Atypical meningiomas represent a minority of intracranial meningiomas yet carry more aggressive biological behavior and higher recurrence rates, making preoperative differentiation essential for optimal management. We report a 56-year-old female presenting with progressive headaches, drowsiness, and vomiting, with no focal neurological deficits. Brain Magnetic Resonance Imaging (MRI) revealed a dural-based lesion at the posterior falx with cystic components, irregular margins, heterogeneous enhancement, disproportionate peritumoral edema, all suggesting atypicality. Subtotal resection followed by histopathological analysis confirmed a atypical meningioma. This case underscores the pivotal role of comprehensive MRI—including conventional sequences and advanced techniques—in identifying features of atypical meningioma even without definitive brain invasion, thereby guiding appropriate surgical planning and consideration of adjuvant therapy.

## Introduction

Meningiomas are among the most common primary intracranial tumors. The majority are benign, but a significant proportion (5%-15%) are classified as atypical, exhibiting a more aggressive biological behavior and higher recurrence rates [1,2]. Preoperative differentiation between benign and atypical meningiomas is clinically significant, impacting surgical strategy and the potential need for adjuvant therapies. Neuroimaging, particularly advanced MRI techniques, has become crucial in identifying subtle characteristics that may suggest a higher grade. This report illustrates a case where comprehensive radiological assessment provided strong preoperative evidence for an atypical meningioma, despite the lack of clear brain invasion, at our institution in Bandung, Indonesia.

## Case presentation

**Fig. 1:**
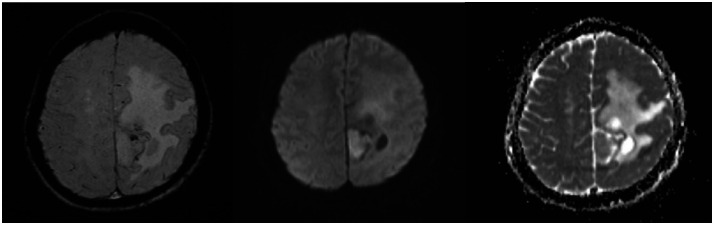
MRI of an atypical meningioma demonstrating areas of susceptibility on SWI (left), diffusion restriction on DWI (center), and corresponding low signal on the ADC map (right).

**Fig. 2:**
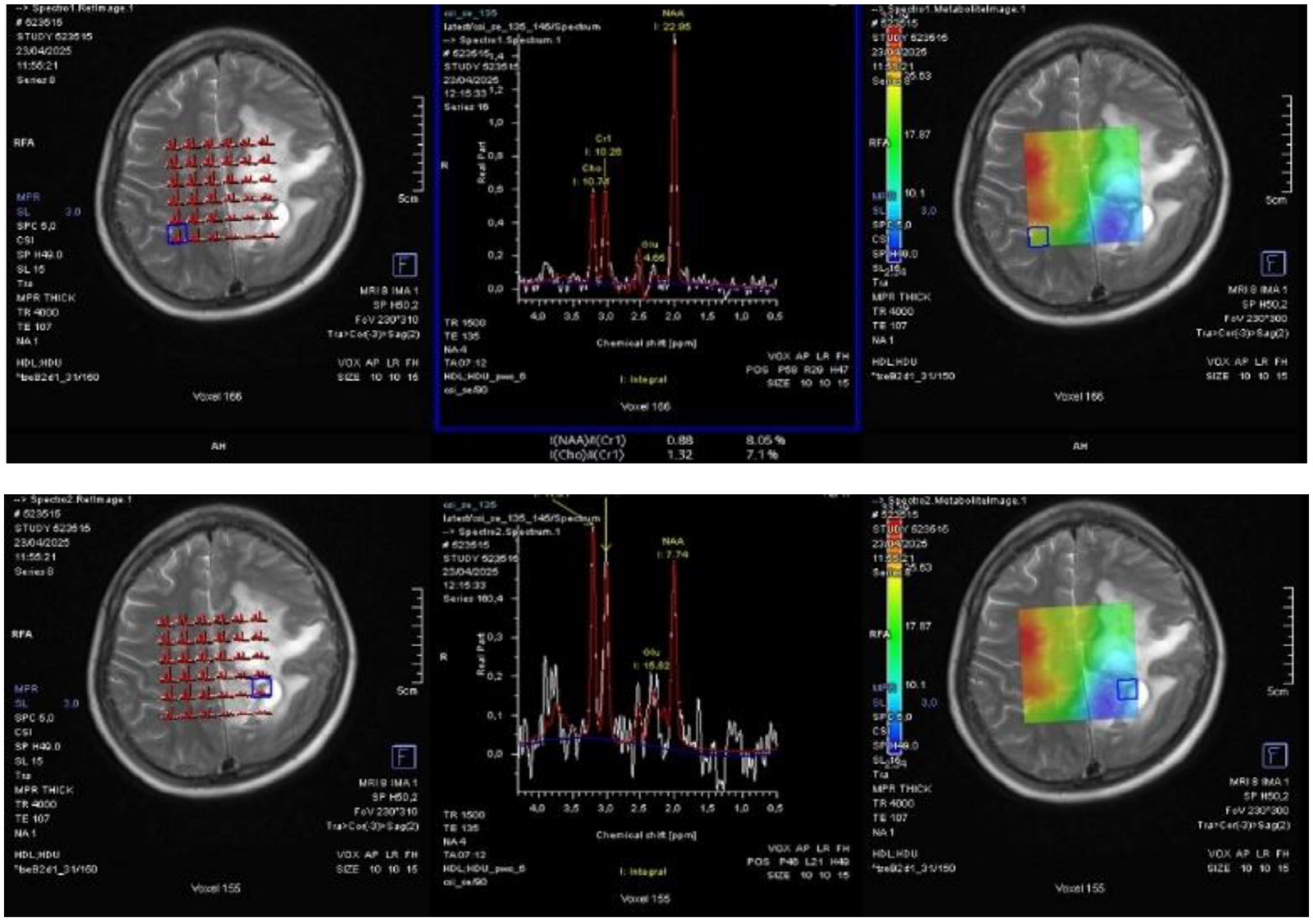
MR spectroscopy shows elevated choline ratio with decreased NAA.

A 56-year-old female presented to the Neurosurgery Outpatient Clinic at Dr Hasan Sadikin General Hospital, Bandung. The primary complaints were progressively worsening headaches over the past one year, which had become constant and severe in the one weeks prior to presentation. The patient appeared drowsy, Glasglow Coma Scale (GCS) was 11. The complaint was also accompanied by two episodes of vomiting. There was no history of limb weakness, seizures, slurred speech, facial asymmetry, behavioral changes, or fever. Motor examination showed no evidence of lateralization. Sensory examination revealed a response to painful stimuli. Physiological reflexes were present bilaterally, and the Babinski reflex was negative on both sides. Initial laboratory investigations were within normal limits. A comprehensive Magnetic Resonance Imaging (MRI) scan of the brain with intravenous gadolinium contrast was subsequently performed a dural-based lesion in the posterior falx cerebri with surrounding cystic components, irregular margins, the lesion partly gives restricted diffusion on Diffusion-Weighted Imaging - Apparent Diffusion Coefficient (DWI-ADC), giving blooming artifacts on Susceptibility-Weighted Imaging (SWI) (Fig. 1). Post-contrast gives strong enhancement. MR spectroscopy demonstrated elevated choline ratio with decreased N-Acetylaspartate (NAA) (Fig. 2). These findings raised suspicion for an atypical meningioma, which was histopathologically confirmed as a World Health Organization (WHO) grade II (atypical) meningioma after subtotal resection [3].

## Discussion

This case highlights the valuable contribution of advanced radiological imaging in differentiating atypical meningiomas from their more common benign counterparts, even in the absence of definitive brain invasion. Preoperative identification of features suggestive of atypicality is critical for optimal patient management.

Conventional MRI sequences offered more granular detail. The heterogeneous enhancement observed on post-contrast T1-weighted images, a deviation from the typical homogeneous enhancement of benign meningiomas, was a significant finding pointing towards atypicality [4]. The disproportionate peritumoral edema on T2-weighted imaging was another prominent feature, strongly correlating with higher tumor grade and increased permeability of the tumor vasculature [5]. While clear brain invasion was not definitively seen, the focally irregular tumor-brain interface still raised a degree of suspicion regarding aggressive growth. The presence of prominent intralesional flow voids also suggested increased vascularity often seen in higher-grade tumors.

The advanced MRI techniques were particularly informative. DWI and ADC maps demonstrating restricted diffusion within the tumor are highly indicative of increased cellularity, a core feature of atypical and anaplastic meningiomas [6,7]. These quantitative imaging biomarkers offer objective data that complement subjective visual assessment.

Despite the absence of definitive brain invasion on imaging or confirmed histologically, the combination of extensive peritumoral edema, heterogeneous enhancement, collectively pointed towards a high probability of atypical meningioma. This comprehensive radiological assessment enabled the neurosurgical team to be well-prepared for a potentially more aggressive tumor, influencing the extent of resection planned and prompting the discussion of adjuvant radiotherapy postoperatively. Even with gross total resection, the atypical grade itself warrants consideration of adjuvant therapy due to the elevated recurrence risk [8].

## Conclusion

This case emphasizes that atypical meningiomas often manifest distinct radiological features, even without overt brain invasion. A meticulous evaluation using conventional MRI, coupled with advanced techniques such as DWI/ADC, can provide crucial preoperative insights into tumor grade, alerting clinicians to the possibility of an atypical meningioma. Recognizing these imaging characteristics is paramount for guiding optimal surgical strategy and ensuring appropriate patient counselling and postoperative management, including the consideration of adjuvant therapies, to improve long-term outcomes for this more aggressive meningioma subtype.

## Patient consent

We confirm that we have obtained written, informed consent from the patient for the publication of this case report. The patient has been thoroughly informed about the details that will be published and understands the implications of the publication.
